# Autoantibodies and B Cells: The ABC of rheumatoid arthritis pathophysiology

**DOI:** 10.1111/imr.12829

**Published:** 2019-12-16

**Authors:** Mikhail Volkov, Karin Anna van Schie, Diane van der Woude

**Affiliations:** ^1^ Department of Rheumatology Leiden University Medical Center Leiden The Netherlands

**Keywords:** autoantibodies, autoimmunity, B cells, rheumatoid arthritis

## Abstract

Rheumatoid arthritis (RA) is an autoimmune disease characterized by joint inflammation. In the last few decades, new insights into RA‐specific autoantibodies and B cells have greatly expanded our understanding of the disease. The best‐known autoantibodies in RA—rheumatoid factor (RF) and anti‐citrullinated protein antibodies (ACPA)—are present long before disease onset, and both responses show signs of maturation around the time of the first manifestation of arthritis. A very intriguing characteristic of ACPA is their remarkably high abundance of variable domain glycans. Since these glycans may convey an important selection advantage of citrulline‐reactive B cells, they may be the key to understanding the evolution of the autoimmune response. Recently discovered autoantibodies targeting other posttranslational modifications, such as anti‐carbamylated and anti‐acetylated protein antibodies, appear to be closely related to ACPA, which makes it possible to unite them under the term of anti‐modified protein antibodies (AMPA). Despite the many insights gained about these autoantibodies, it is unclear whether they are pathogenic or play a causal role in disease development. Autoreactive B cells from which the autoantibodies originate have also received attention as perhaps more likely disease culprits. The development of autoreactive B cells in RA largely depends on the interaction with T cells in which HLA “shared epitope” and HLA DERAA may play an important role. Recent technological advances made it possible to identify and characterize citrulline‐reactive B cells and acquire ACPA monoclonal antibodies, which are providing valuable insights and help to understand the nature of the autoimmune response underlying RA. In this review, we summarize what is currently known about the role of autoantibodies and autoreactive B cells in RA and we discuss the most prominent hypotheses aiming to explain the origins and the evolution of autoimmunity in RA.

## 1 INTRODUCTION

Rheumatoid arthritis (RA) affects approximately 1% of the population and is a chronic autoimmune disease. It is characterized by joint inflammation that leads to cartilage and bone damage and, if left untreated, can potentially lead to disability. In the majority of patients, RA is a chronic, lifelong disease in which the goal of treatment is to reach and maintain low disease activity or, ideally, remission (ie, the absence of disease activity). Despite the fact that recent advances in therapy have significantly improved patients’ clinical outcomes and quality of life, the underlying autoimmunity remains and necessitates long‐term treatment, as tapering and/or withdrawal often lead to clinical relapse.

The joint tissue (synovium) in RA patients is typically infiltrated by immune cells such as T cells, B cells, and macrophages which produce a variety of proinflammatory cytokines facilitating inflammation and eventually leading to tissue destruction. The origin of the autoimmune response in RA has proven difficult to unravel, although epidemiological research has succeeded in identifying both genetic and environmental factors contributing to the risk of RA.

Immunologically, RA can be subdivided into two main categories: seropositive and seronegative RA, based on the presence of autoantibodies, mainly rheumatoid factor (RF) and anti‐citrullinated protein antibodies (ACPA). The presence of these autoantibodies can be seen in 50% of early RA patients and in up to 80% of patients with established RA.^1^ This is explained by the fact that seronegative disease is more transient: Many RF‐ and ACPA‐negative patients do not develop a chronic progressive disease, and therefore, cohorts following patients over a long period of time retain mostly seropositive patients. Seropositive RA is also associated with more severe disease and worse clinical outcomes.^2^, ^3^ This led to the idea that autoantibodies or the underlying autoreactive B and T cell responses may play an important role in the pathogenesis of RA.

Studies published in the last few years have broadened the existing concepts and provided novel insights into the etiology and mechanisms of autoimmunity in RA. In this review, we provide a summary of what is currently known about the routinely used immune biomarkers of RA, such as RF and ACPA. The latter, being the most prominent RA‐specific autoantibody, is discussed in more detail, including its specific features and pathogenicity. We also provide an overview of the newly discovered autoantibodies targeting various posttranslational modifications, as well as their relatedness with ACPA and each other. Finally, we discuss the most prominent hypotheses aiming to explain how autoimmunity in RA may start and which factors play an important role in the evolution toward disease.

## 2 RHEUMATOID FACTOR

RF is the most well‐known autoantibody in RA, and it is included in major classification criteria, such as 1987 ACR and 2010 ACR/EULAR classification criteria. RF is defined as a class of immunoglobulins (Ig‐s) directed against the Fc region of IgG. Different isotypes of RF can be detected, of which IgM is the most prevalent.^4^ The detection of RF is traditionally used in clinical settings to differentiate RA from other diseases with similar symptoms. Although various titers and isotypes of RF can be seen in a vast range of different diseases,^4^ high titers of IgM and IgA are thought to be highly indicative of RA.^5^ Whether RF levels also correlate with clinical disease activity is a matter of debate: Its levels fluctuate in early RA independently of clinical activity,^6^ and decrease in RF levels upon treatment is not necessarily associated with clinical response.^7^ Nevertheless, a decrease in RF levels can often be seen in patients effectively treated with disease‐modifying anti‐rheumatic drugs (DMARDs).^4^ Although these level fluctuations may be interesting from a scientific point of view, they are generally not considered meaningful in daily clinical practice.

Despite the fact that RF has been known for decades, its role in RA pathogenesis is still unclear. However, it is likely that RF can exert its pathogenic properties via immune complex formation. Under healthy circumstances, RF can form complexes with IgG bound to antigens to facilitate the immune response.^8^, ^9^ In RA however, RF shows signs of affinity maturation, which is not observed in healthy individuals.^10^ Possibly, the high titers of high‐affinity RF found in synovial fluid of RA patients lead to a derailed facilitating function, thus perpetuating inflammation. RF immune complexes are thought to promote inflammation by stimulating the production of proinflammatory cytokines, such as tumor necrosis factor (TNF) α.^11^

Overall, the exact role of RF in RA development appears to be enigmatic and its presence in other diseases highlights RF as a rather general autoimmunity‐related phenomenon. Despite this, RF remains a useful diagnostic marker of RA used in daily clinical practice.

## 3 ANTI‐CITRULLINATED PROTEIN ANTIBODIES

Over the last decades, ACPA have been at the center of attention of autoantibody research due to their high specificity for RA (ACPA are hardly ever found in individuals without RA).^12^ These autoantibodies are directed against citrulline residues on proteins or peptides. Citrullination (or deimination) is an irreversible posttranslational modification of arginine that is mediated by enzymes called peptidyl arginine deiminases (PADs). The putative mechanisms of how citrullination may lead to a breach of tolerance in RA are reviewed in greater detail below.

ACPA often co‐occur with RF and are present in approximately 50%‐60% of early RA patients and 60%‐90% of patients with established disease.^12^, ^13^, ^14^ Only 1%‐3% of healthy individuals are positive for ACPA, and mostly at low levels.^15^ ACPA are substantially more specific for RA than RF (95% for ACPA and 85% for RF^16^), which makes them a prominent RA biomarker. ACPA have therefore been included in the widely used ACR‐EULAR 2010 classification criteria.^17^ On the one hand, the presence of ACPA can be observed for years in asymptomatic individuals and does not necessarily lead to RA development.^18^ On the other hand, in individuals suffering from musculoskeletal symptoms including joint pain (arthralgia), the presence of ACPA is predictive of RA development^19^: A positive predictive value of more than 60% was observed in individuals with recent‐onset clinically suspect arthralgia.^20^ ACPA are furthermore a hallmark of seropositive RA, which is associated with higher disease severity, radiographic joint damage,^21^, ^22^, ^23^, ^24^, ^25^ and the occurrence of extra‐articular manifestations, such as cardiovascular^26^ and pulmonary involvement.^27^ Interestingly, ACPA were shown to be associated with cardiovascular mortality even in the absence of RA.^28^

**Figure 1:**
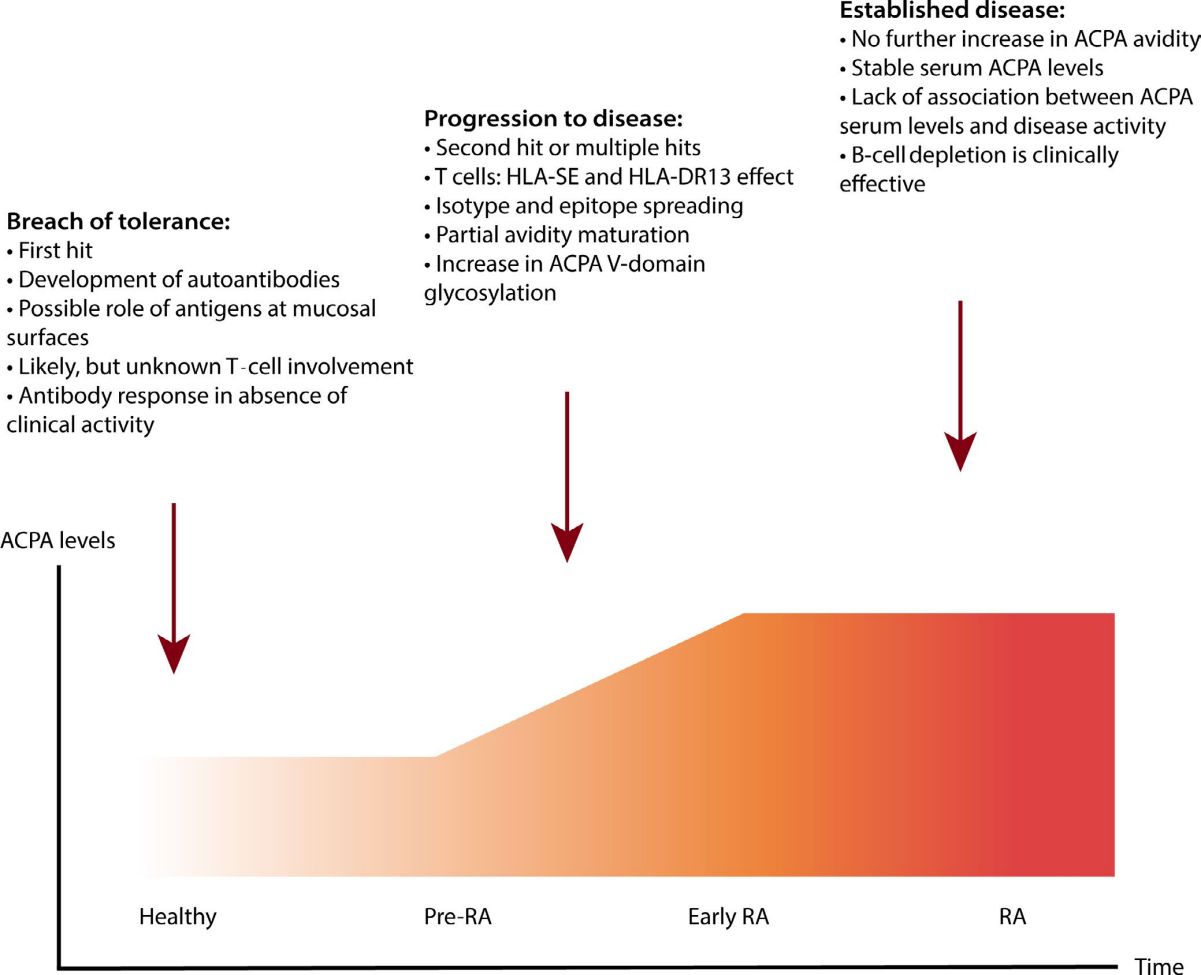
Evolution of RA and development of autoimmunity. Note: The graph summarizes current evidence and prominent hypotheses linking development of autoimmunity and clinical symptoms in patients with rheumatoid arthritis. The first signs of the pathological process manifest years before the disease onset in the form of autoantibody positivity, including anti‐citrullinated protein antibodies (ACPA), anti‐carbamylated protein antibodies, and rheumatoid factor. The autoantibodies are class‐switched, which suggests T cell help, however, autoantibody positivity does not necessarily lead to disease onset. Close to disease onset, the ACPA response matures and accumulates more variable domain glycosylation sites. This process seemingly depends on HLA and T cells with some role for HLA shared epitope (HLA‐SE) and HLA‐DR13. In established disease, the ACPA response remains stable, while B cell depletion is clinically effective

As mentioned, ACPA can be found years before the RA onset and its presence does not necessarily lead to RA development (Figure 1). Many studies have therefore investigated specific ACPA characteristics presuming that perhaps a distinct type of ACPA or a certain ACPA feature that develops during the maturation of the immune response could predict disease onset. For instance, substantial amount of research has been dedicated to find out the most important antigen(s) recognized by ACPA, in hope of finding a “predictive antigen.” However, diagnostic assays for ACPA detection have employed various cyclic citrullinated antigens,^29^, ^30^, ^31^, ^32^ indicating that the presence of citrulline—but not one specific citrullinated epitope—is required for recognition by ACPA. The neighboring position of glycine resulting in the Cit‐Gly motif has been shown to be preferential, but not obligatory for recognition by ACPA.^33^, ^34^ Consequently, many citrullinated proteins have been shown to be recognized by ACPA, for example, fibrinogen,^35^ vimentin,^36^ α‐enolase,^37^ and collagen type II.^38^ Initially, there were reports suggesting a specific role in disease development for particular citrullinated proteins, such as vimentin and/or α‐enolase.^39^, ^40^ Importantly, these findings were later not confirmed when studied in pre‐RA individuals.^41^ In addition, defined ACPA fine specificities showed no correlation with clinical activity and radiographic damage in RA.^42^, ^43^

Instead, the current consensus in the field suggests that not a particular ACPA specificity, but the epitope spreading together with increased isotype usage represent the important markers of disease progression. These phenomena, together with a rise in ACPA levels, have been found to occur shortly before disease onset.^44^, ^45^

**Figure 2:**
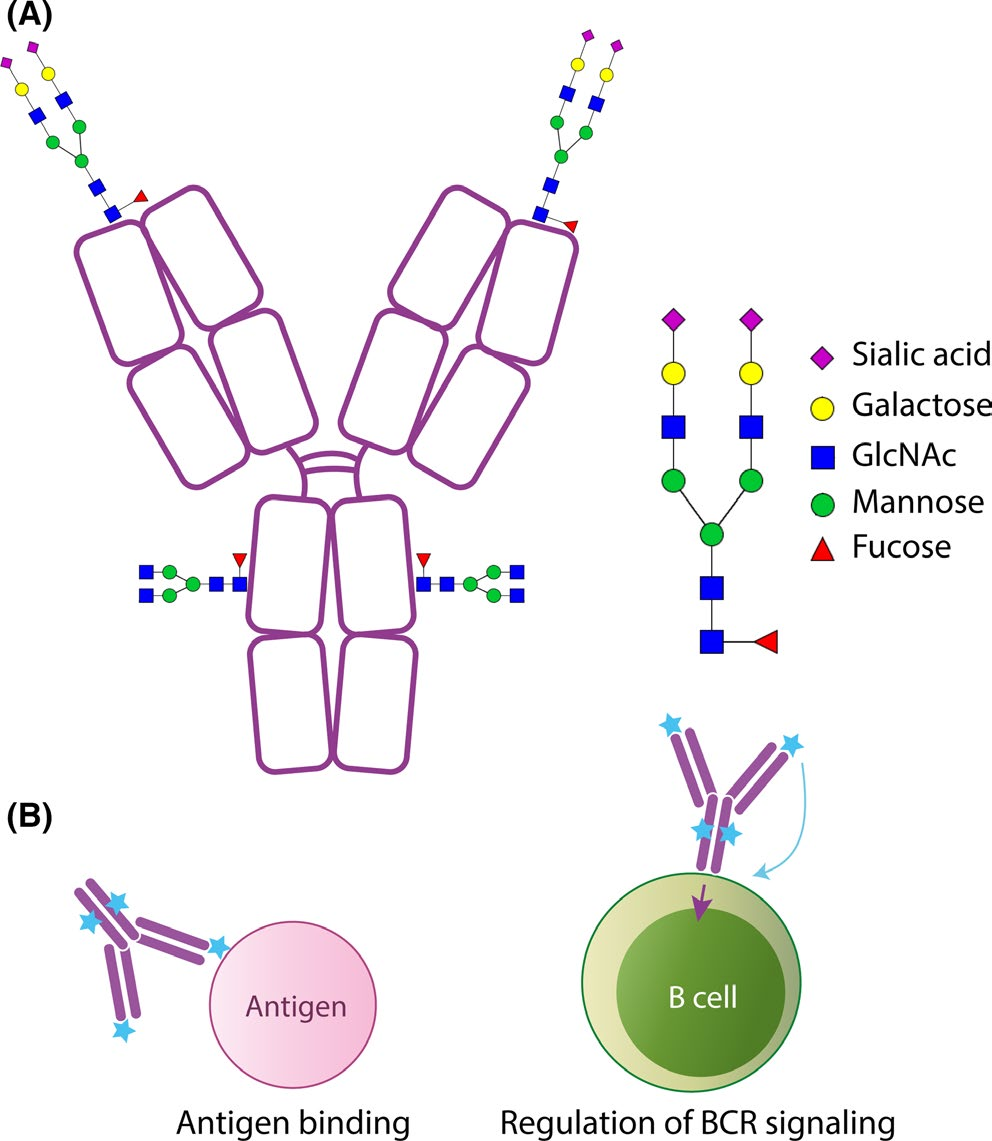
Variable domain glycosylation of ACPA. Note: All human IgG molecules bear glycosylation sites in the constant domain of their heavy chains. Anti‐citrullinated protein antibody IgG molecules are also highly glycosylated in their variable domain (V‐domain). These V‐domain glycans are structurally different from the constant domain glycans and bear higher abundance of galactosylation and sialylation. The functional properties of the V‐domain glycans are not yet known; however, they may affect affinity and thus impact antigen binding. Another possible function of these glycans is their influence on B cell receptor signaling, which may provide a survival advantage for the citrulline‐reactive B cells. This effect may be achieved by either change in BCR signaling threshold or binding to lectins expressed by other cells or the B cells themselves

Another interesting observation regarding ACPA is that they have a larger molecular weight as compared to most antibodies, due to the fact that they carry N‐linked glycans in their variable domains (Figure 2A).^46^ *N‐*linked glycosylation of proteins (including IgG) requires the presence of a certain consensus sequence, (N‐X‐S/T; where X is not a proline). The IgG heavy chain sequence is known to have one highly conserved *N‐*glycosylation site at position 297, which results in the presence of one glycan in the constant domain of each IgG heavy chain. The IgG variable domain can also contain glycosylation sites, which is found in ~18% of V_H_ sequences.^47^ Although the presence of a glycosylation site in the DNA sequence does not necessarily lead to glycosylation of the protein, the large majority of IgG V‐domain glycosylation sites indeed seems to be glycosylated on the protein level. For total IgG, V‐domain glycosylation has been estimated to be around 14%.^48^ For citrulline‐reactive B cells, however, over 80% of the sequences were found to contain V‐domain glycosylation sites.^49^ On the protein level, over 90% of ACPA were found to bear V‐domain glycans.^50^ Moreover, increased glycosylation could be seen up to 15 years prior to disease manifestation and increased closer to symptom onset.^51^ Interestingly, ACPA V‐domain glycosylation may also have clinical relevance, as ACPA‐positive predisease individuals were more prone to develop RA when their ACPA was highly V‐domain glycosylated.^52^ V‐domain glycosylation could therefore possibly serve as future biomarker to predict RA development in predisease individuals. The possible role of V‐domain glycosylation in the biology of citrulline‐reactive B cells is discussed below.

### 3.1 ACPA and HLA

Human leukocyte antigen (HLA) is known to be the most important genetic risk factor in RA. In particular, a common amino acid sequence at position 70‐74 of the HLA‐DRB1 molecule (the so‐called “shared epitope,” HLA‐SE) was shown to be associated with disease.^53^ Notably, HLA‐SE alleles are only associated with ACPA‐positive RA, but not with ACPA‐negative RA,^54^ which indicates a strong link between HLA‐SE and the ACPA response in RA patients. ACPA positivity in the absence of RA is a rare phenomenon; however, in ACPA‐positive healthy individuals, the presence of ACPA was not associated with HLA‐SE alleles, indicating that the HLA‐SE alleles are not associated with ACPA as such, but rather with ACPA‐positive disease.^55^, ^56^ In contrast to HLA‐SE, other alleles of HLA‐DRB1 (namely, HLA‐DRB1*13) were shown to be protective for RA development. The mechanisms of how HLA‐SE and HLA‐DRB1*13 could be involved in disease pathogenesis are discussed below.

### 3.2 Pathogenicity of ACPA

The actual role of ACPA in RA pathogenesis is a matter of debate, and several hypotheses have been put forward. As inflammation is key in RA pathogenesis, a lot of attention has been focused on the idea that ACPA could activate immune cells and upregulate production of proinflammatory cytokines. Indeed, in mouse models, murine and passively transferred human ACPA were reported to enhance arthritis.^57^, ^58^ Similar to immune complexes containing recall antigens, immune complexes containing ACPA have been shown to induce TNFα secretion in monocytes and macrophages via FcγRII,^59^, ^60^ while another study has highlighted the potential importance of FcγRI and neutrophil activation.^61^ IgM and IgA RF have been proposed to potentiate this ACPA‐mediated secretion of proinflammatory cytokines as well as complement activation.^62^ ACPA by themselves have furthermore been shown to activate complement not only via the classical pathway, but also via the alternative pathway.^63^

The key feature of RA is cartilage and bone damage in the form of erosions. Not surprisingly, substantial research has focused on the link between ACPA and activation of osteoclasts, the cells driving the erosive process in the bone. One scenario explaining this is indirect activation mediated by interaction of ACPA‐containing immune complexes with FcγRs and release of TNFα, as discussed above. Besides that, it was hypothesized that ACPA could have an agonistic effect and enhance osteoclastogenesis by directly binding citrullinated proteins on the surface of osteoclasts. Several studies have contributed to this idea by reporting ACPA‐specific stimulation of osteoclast differentiation and enhancement of bone resorption *ex vivo* and in a mouse model.^64^, ^65^, ^66^ In one of these studies, the direct effect of ACPA potentiating osteoclast differentiation was shown using polyclonal ACPA isolated from patients and ACPA monoclonal antibodies; however, some of the monoclonal antibodies were later shown to lack citrulline specificity.^67^ The fact that these monoclonal antibodies did not have to be ACPA to stimulate osteoclastogenesis greatly complicates the interpretation of the results and indicates that the described phenomena may in fact be independent of the antibody specificity.

In conclusion, ACPA demonstrate normal properties of antibodies in terms of being able to activate immune cells and complement via their Fc‐regions. The idea of ACPA having a unique ability to interact with osteoclasts via their variable domain regions is intriguing; however, the data published so far appear to be controversial. Overall, the pathogenicity of ACPA and the mechanisms involved in it remain a matter of debate, which needs to be resolved by future studies.

## 4 ANTI‐CARBAMYLATED PROTEIN ANTIBODIES

Carbamylation (or homocitrullination) has become the second discovered posttranslational modification that is recognized by an autoantibody response in RA. The antibodies against carbamylated proteins received the name “anti‐CarP.” Carbamylation is a posttranslational non‐enzymatic reaction mediated by cyanate, resulting in the conversion of lysine into carbamyl‐lysine (or homocitrulline). Cyanate is in chemical equilibrium with urea, and only a low level of cyanate can be observed at normal conditions. However, in certain conditions, such as smoking, inflammation, and renal failure, cyanate levels increase leading to enhanced carbamylation.^68^

Anti‐CarP tend to be found mainly in ACPA‐positive RA patients, but is also present in 8%‐14% of ACPA‐negative patients.^69^ Similar to ACPA, anti‐CarP can also be present years before disease onset.^70^ Furthermore, the anti‐CarP response shows isotype switching and is, like the ACPA response, of overall low avidity as compared to recall antigens.^71^

## 5 ANTI‐ACETYLATED PROTEIN ANTIBODIES

Acetylation is a reaction leading to the most recently discovered posttranslational modification recognized by autoantibodies of RA patients. There are two types of protein acetylation known so far: N‐terminal acetylation, an irreversible enzymatical process occurring at the N‐terminus of the polypeptide, and lysine acetylation, a reversible process converting lysine residues to acetyllysines. Lysine acetylation in eukaryotes is enzymatic, whereas in bacteria it can also occur non‐enzymatically in the presence of acetyl‐CoA.^72^ Among these two types of acetylation, autoantibodies of RA patients seem to recognize the acetyllysines. Anti‐acetylated protein antibodies (AAPA) against an acetylated vimentin peptide were found to be present in 40% of RA patients, largely confined to the ACPA‐positive subgroup.^73^

The link between acetylation and autoantibodies is especially intriguing as bacteria are known to not only acetylate their own proteins, but also modify host proteins.^74^, ^75^ This provides a potential mechanism by which bacteria can trigger breach of tolerance toward modified self‐proteins.

## 6 ANTI‐MAA AND ANTI‐MDA ANTIBODIES

**Figure 3:**
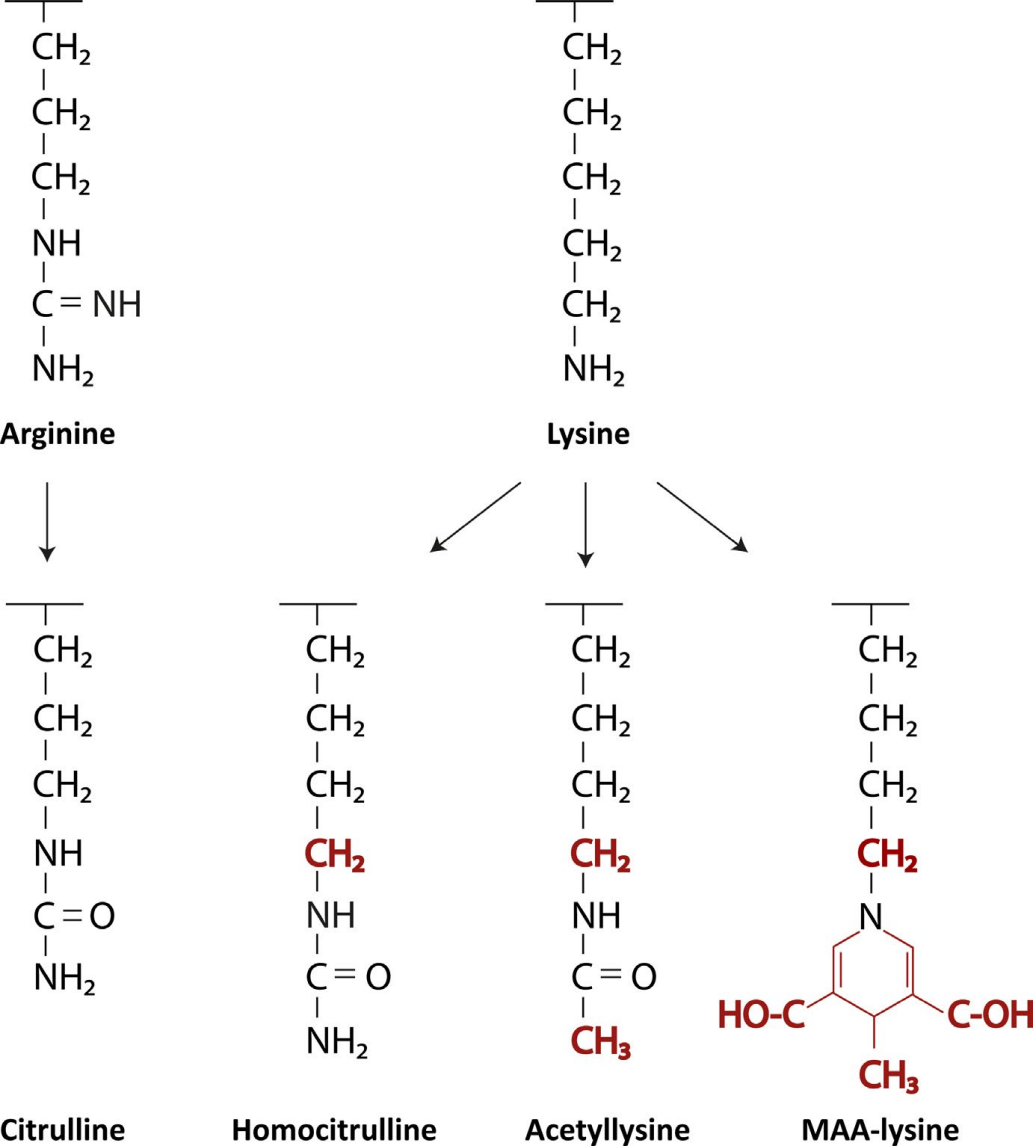
Posttranslational modifications targeted by autoantibodies in RA. Note: Citrulline is an enzymatically generated posttranslational modification (PTM) of arginine, while homocitrulline, acetyllysine, and malondialdehyde‐acetaldehyde (MAA)‐lysine are thought to be products of non‐enzymatic processes. Interestingly, all listed PTMs have neutral charge, as opposed to the positive charge of the original arginine and lysine. Homocitrulline and acetyllysine, but not MAA‐lysine, bear evident structural similarity to citrulline. The differences in structure between citrulline and other PTMs are marked in red

Malondialdehyde (MDA) is a product of lipid peroxidation that can be adducted to lysine residues of proteins. Through a reaction with acetaldehyde, MDA can be further modified to form a more stable malondialdehyde‐acetaldehyde (MAA) adduct. These modifications have been associated with inflammation and more specifically with atherosclerosis.^76^ An interesting aspect of MAA and MDA is their high immunogenicity, which implies their potential role as an (auto)antigen.^77^ Anti‐MAA antibodies are associated with coronary artery disease^78^ and are furthermore found in RA patients, mainly but not exclusively within the seropositive group.^79^ MAA (the antigen) can also be found in higher concentrations in lung tissue of RA patients with interstitial lung disease (ILD) as compared to ILD patients without RA.^80^ Unlike AAPA and anti‐CarP, anti‐MAA were not shown to be cross‐reactive with ACPA (discussed below), which could indicate that this response is less related to the other AMPA (Figure 3).^79^ Anti‐MAA antibodies have furthermore been reported in patients with osteoarthritis, systemic lupus erythematosus, and even alcohol use disorders, indicating that they lack RA specificity.^81^, ^82^

## 7 CLINICAL RELEVANCE OF AUTOANTIBODIES IN RA

Since the discovery of these autoantibodies, various studies have investigated their implication in diagnostics as well as their association with clinical phenotypes and disease outcomes. The value of determining various autoantibodies appears to be remarkably different depending on what phase of the disease is studied. In at‐risk individuals without arthralgia, triple positivity for autoantibodies (RF, ACPA, and anti‐CarP) has a higher association with disease development.^83^ Moreover, in a meta‐analysis investigating various cohorts of presymptomatic individuals, triple positivity resulted in high specificity (98%‐100%), but low sensitivity (11%‐39%) for prediction of RA development.^84^ However, in individuals who have already progressed to the stage of arthralgia, anti‐CarP did not have an additive value to ACPA and RF in predicting arthritis onset.^85^ Once disease has developed, the value of anti‐CarP regains its importance: The presence of anti‐CarP has been independently associated with higher disease activity at baseline and over time,^86^, ^87^ as well as with radiographic damage.^88^, ^89^ At baseline, a broad autoantibody profile regarding different isotypes of RF, ACPA, and anti‐CarP is associated with early treatment response; but the importance of this broad profile diminishes over time.^90^ Intensity of treatment correlates with a decrease in autoantibody levels and reflects the level of immunosuppression, although this detectable decrease has only limited relevance for clinical activity and long‐term treatment response.^91^ These observations shed light on the possible role of autoantibodies in (pre‐)RA patients, although their exact functions need to be elucidated in future studies.

## 8 FROM ANTIBODIES TO B CELLS

An exceeding amount of research regarding the citrulline‐reactive immune response so far has been focused on its humoral component. Importantly, it is unclear what role citrulline‐reactive B cells play. Over the last decade, therapies targeting B cells—such as the anti‐CD20 therapeutic antibody rituximab—have proven effective in RA, indicating a potential role for (autoreactive) B cells in disease pathogenesis.^92^ Intriguingly, clinical efficacy does not seem to be closely linked with detectable changes in AMPA profiles, and even at low disease activity, patients often remain autoantibody‐positive.^6^, ^93^, ^94^ Although it remains possible that anti‐CD20 treatment induces yet unidentified changes in the autoantibody response, these findings suggest that autoantibodies themselves may have a limited role in driving the disease. Whereas the clinical efficacy of CD20‐depletion points toward a role for B cells in RA pathogenesis, the role of plasma cells in RA remains to be elucidated. Treatment directly targeting plasma cells, such as anti‐CD38 agents or proteasome inhibitors, has not been so far investigated in RA.

In rituximab‐naive RA patients, the frequency of citrulline‐reactive B cells in the peripheral blood generally correlates with ACPA titers and varies greatly with a median of ~ 1 in 12,500 B cells bearing B cell receptors (BCRs) reactive to citrullinated peptides/proteins.^95^ Most of these cells are class‐switched postgerminal center memory B cells expressing IgG or IgA BCRs. In addition, a proportion of ACPA expressing cells (~7%) are plasmablasts or plasma cells.^96^ These ACPA plasmablasts may migrate to the synovial compartment where they encounter a microenvironment that supports their long‐term survival and stimulates them to secrete ACPA.^97^, ^98^ Alternatively, citrulline‐reactive B cells could migrate to synovium at an earlier stage of development and undergo differentiation in ectopic lymphoid structures located in RA synovium.^99^, ^100^ It is conceivable that the memory pool of citrulline‐reactive B cells is directly renewing these plasmablasts/plasma cells in an ongoing manner. This is supported by the fact that IgM ACPA is found in sera of both early undifferentiated arthritis and established RA patients.^101^, ^102^ This observation is important, since the IgM half‐life is only 5‐8 days^103^ and IgM secreting plasma cells are known to be mostly short‐lived.^104^ Together, this suggests a continuous activation and recruitment of citrulline‐reactive B cells. These findings imply a striking difference between the ACPA response and more classical immune responses, in which B cells have prolonged periods of quiescence due to the absence of antigens. As posttranslational modifications are ever‐present antigens, the ACPA response may be continuously triggered, leading to proliferation and differentiation of citrulline‐reactive memory B cells

## 9 HYPOTHESES ELUCIDATING MECHANISMS OF AUTOIMMUNITY IN RA

In this section, we will discuss the most prominent hypotheses aiming to explain how autoimmunity in RA may be induced, maintained, and how it can subsequently evolve toward disease. First, we will elaborate on what could be the source of the antigens triggering the breach of tolerance. In particular, we will review the potential roles of smoking and the microbiome. Second, we will discuss how autoreactive B cells in RA may evade tolerance checkpoints and maintain the autoimmune response. Finally, we shift focus to T cells and HLA class II and review their role in stimulating B cells leading toward disease development.

### 9.1 Antigens initiating breach of tolerance against citrullinated proteins

Despite the extensive research in this field, it remains unknown what antigen (or antigens) is the initial target of the citrulline‐reactive response that results in ACPA development. These citrullinated antigens could be derived from self‐ or non‐self‐proteins. Besides that, they may not even have to be citrullinated, as the citrulline‐reactive B cells may in fact be cross‐reactive. This opens up the possibility that citrulline‐reactive B cells could get activated as a result of recognizing an antigen containing a homocitrulline or an acetyllysine, which could again be derived from self‐ or non‐self‐proteins. This represents a complex puzzle for which at the moment there is no conclusive answer.

In the following paragraphs, we will focus on some prominent hypotheses describing how antigens could lead to the breach in tolerance and review their current status based on the published data.

### 9.2 Citrullination in health and disease

PADs convert arginine into citrulline in a calcium‐dependent manner and thereby cause changes in protein properties.^105^ Physiologic citrullination plays a role in apoptosis, regulation of gene expression, and organization of structural cell proteins.^106^ However, in the presence of high calcium levels, citrullination may become less controlled and lose specificity.^107^ Thus, for many proteins found to be citrullinated in human, it is unknown whether citrullination has a physiological function or happens erratically. In RA, citrullination is thought to have a distinct pattern potentially affecting protein sites that would not have become citrullinated under healthy conditions and thus generates neoepitopes in abnormally hypercitrullinated molecules. These neoepitopes may be then recognized as antigens by the immune system and evoke an antibody response.^108^ Several mechanisms are thought to contribute to this hypercitrullination mediated by human PADs. These mechanisms include cell death pathways, such as NETosis, necrosis, and leukotoxic hypercitrullination, all of which could be mediated by environmental factors, such as smoking, bacterial, or/and immunological phenomena. These processes are accompanied by membrane damage, resulting in calcium influx, activation of PADs, and subsequent hypercitrullination.^108^ Another, completely different mechanism of citrullination is mediated by an extrinsic, bacterial PAD enzyme, synthesized by a periodontitis‐causing pathogen called *Porphyromonas gingivalis* (*Pg*).^109^ There have been several hypotheses aiming to explain how this abnormal citrullination is triggered by environmental factors, among which the most prominent refer to smoking and bacterial involvement.

### 9.3 Smoking and citrullinated antigens

So far, cigarette smoking has been the most consistently described environmental risk factor for developing RA and is most strongly associated with seropositive RA.^110^, ^111^, ^112^, ^113^, ^114^ Cigarette smoke extract can interact with synovial fibroblasts from RA patients, leading to upregulation of proinflammatory cytokines.^115^ Moreover, smoking has been thought to be linked to citrullination, especially after it was shown that smoking associates with higher expression levels of the PAD2 enzyme, leading to increased citrullination levels in the lung.^116^ In epidemiological studies, smoking was found to be associated with HLA‐SE positivity in RA patients,^117^ which implied a possible link with ACPA. Subsequently, an association between smoking and ACPA positivity was demonstrated,^118^, ^119^ and this association could be mostly observed in individuals positive for HLA‐SE.^120^, ^121^, ^122^ This interaction between the environmental risk factor smoking and the genetic risk factor HLA‐SE has given rise to a prominent hypothesis about RA pathogenesis^123^, ^124^: Smoking leads to inflammatory changes in lungs subsequently causing activation of PADs and generation of citrullinated residues in the lung tissue. These citrullinated proteins are then recognized by citrulline‐reactive B cells, which present these citrullinated antigens to HLA‐SE‐restricted T cells and thus get help to undergo further differentiation. As a result of these events, tolerance toward citrullinated proteins is lost, after which the immune response targets citrullinated proteins located in synovial tissue and triggers the onset of arthritis.

The idea of directly linking smoking with the breach of tolerance against citrullinated proteins is of high interest; however, this relationship may be more complicated. In several more recent studies, the association between smoking and ACPA was not specific for ACPA, but found to be largely due to the co‐occurrence of ACPA and RF, as smoking is associated with RF rather than ACPA^125^, ^126^ or with double positivity alone.^127^ In ACPA‐positive patients with lung disease and without signs of joint inflammation, smoking was not associated with higher ACPA titers or transition toward RA.^128^ This indicates that smoking might not be the unique factor linking lung inflammation with ACPA production and RA development. Future studies are expected to unravel the exact mechanisms underlying these observations.

### 9.4 Microbiome and citrullinated antigens

Another environmental factor associated with RA is mucosal inflammation. So far, periodontitis has been the most prominent topic in this field. RA and periodontitis have been shown to be associated with each other in many clinical and epidemiological studies (extensively reviewed by Potempa et al^129^). Importantly, periodontitis and RA share several genetic and environmental risk factors including HLA‐DRB1 expression, smoking, aging, and socioeconomic status.^129^ There is, however, a study that could not find an association between established RA and periodontitis,^130^ and it is also unclear whether there is a specific causal relationship between the two. On the one hand in this chicken‐or‐egg debate, two meta‐analyses have highlighted RA as—in epidemiological terms—the causal factor by showing increased incidence of periodontitis in RA patients as compared to healthy individuals.^131^, ^132^ On the other hand, periodontal disease was shown to correlate with new‐onset rheumatoid arthritis,^133^ indicating that periodontitis would be the causative agent. Considering the mechanism behind the potential influence of periodontitis on RA pathogenesis, there are a few distinct hypotheses. In these hypotheses, two periodontitis‐causing pathogens, *Porphyromonas gingivalis* and *Aggregatibacter actinomycetemcomitans*, come to the foreground.

*Porphyromonas gingivalis (Pg)* is a unique pathogen in the sense that it has its own PAD enzyme capable of citrullination—PPAD. It is hypothesized that PPAD generates citrullinated neoepitopes that lead to the breach of tolerance toward citrullinated (self)proteins. This is linked to the fact that PPAD works differently from human PAD enzymes: It citrullinates proteins at their C‐terminus, while human PADs act outside the C‐terminus.^134^ On the one hand, this suggests that the immune system may not be inherently tolerant against the epitopes citrullinated by PPAD; on the other hand, the proteins citrullinated by PPAD have not yet been shown to be recognized by human ACPA (extensively reviewed by Gómez‐Bañuelos et al^135^). In a murine model, *Pg* indeed increased citrullination at the site of infection, induced autoantibody production, and led to earlier onset of collagen‐induced arthritis.^136^ Nevertheless, in humans, levels of PPAD‐mediated citrullination were similar in periodontitis patients with and without RA.^137^ Anti‐PPAD antibody levels were furthermore not increased in RA patients with periodontitis as compared to healthy individuals.^138^ This indicates that, although high PPAD activity may increase the presence of citrullinated (auto)antigens, this does not necessarily lead to the breach of tolerance.

For *Aggregatibacter actinomycetemcomitans* (*Aa*), an intriguing hypothesis was suggested as follows: A unique pore‐forming toxin synthesized by this bacterium was shown to upregulate citrullination in neutrophils by human PADs. This would lead to hypercitrullination and cell death, resulting in the release of substantial amounts of citrullinated epitopes in the extracellular environment, which on their turn could breach tolerance.^139^ The same study also suggested that in RA patients, the relationship between HLA‐SE alleles and the presence of ACPA is limited to the individuals exposed to *Aa*. However, this latter observation was not reproduced when tested in another cohort.^140^

For a long time, the gut has been hypothesized to be related to RA pathogenesis, but it remains a highly challenging topic to study. In mice, the K/BxN model of autoimmune arthritis appears to be greatly influenced by the gut microbiota via T follicular helper cells.^141^, ^142^ In SKG mice, another animal model or RA, fecal transfer from RA patients exacerbated the disease stronger as compared to transfers from healthy individuals.^143^ In humans, sequencing studies showed differences in the gut microbiome of RA patients and healthy individuals,^144^ with a special focus on the bacterium *Prevotella copri* (*Pc*), which was found to be relatively abundant in the gut of early RA patients.^143^, ^145^ *Prevotella* species were furthermore found to be abundant in the gut of at‐risk individuals.^146^ Although it was hypothesized that one of *Pc's* antigens could be citrullinated and targeted by ACPA,^147^ at the moment there is a lack of conclusive data linking *Pc* with AMPA.

Overall, the data on RA and microbiome published in the last few years have provided captivating hypotheses and findings. Nonetheless, the data collected so far remain inconclusive; it is also unclear whether there is a specific culprit pathogen or the mechanism of microbiome involvement in RA could be more general and include multiple heterogeneous bacteria.

### 9.5 Antigens containing other modifications: AMPA concept

The three posttranslational modifications described above (citrullination, carbamylation, and acetylation) have a lot in common. First, they share a striking structural similarity (Figure 3), especially citrulline and homocitrulline, which differ by only one CH_2_ group. Acetyllysine is more distinct from citrulline, as besides the extra CH_2_ group, the end amine group is replaced by a methyl group. Second, ACPA, anti‐CarP, and AAPA tend to be present in the same individuals.^69^, ^73^ Interestingly, mice immunized with a carbamylated protein develop antibodies recognizing both carbamylated and acetylated (self) proteins; the same can be observed when mice are immunized with an acetylated protein.^148^ This cross‐reactivity is also detected in patients in which anti‐CarP and ACPA responses show cross‐reactivity to varying degrees.^148^, ^149^, ^150^ Furthermore, preliminary data illustrated that an ACPA/AAPA monoclonal antibody can be cross‐reactive,^151^ and commercially available (polyclonal) antibodies are often unable to distinguish between different citrullinated and carbamylated residues.^152^ Together, these observations hint toward a cross‐reactive antibody response targeting citrullinated, carbamylated, and acetylated proteins.

Due to the cross‐reactivity and evident relatedness of these antibody responses, it seems possible to unite them under the name of “anti‐posttranslationally modified protein antibodies,” or AMPA. It is plausible that the ACPA response may in fact originate from the response that initially targeted another modification, for example, homocitrulline or acetyllysine. In pre‐RA patients, this response may be skewed due to exposure to citrullinated antigens, which can subsequently lead to disease development.

Interestingly, smoking has been found to be linked with other posttranslational modifications besides citrullination, potentially contributing to the development of AMPA. In particular, mice exposed to tobacco smoke show increased carbamylation of vimentin and develop higher titers of antibodies against it.^153^ Smoking can also be indirectly linked to acetylation, as smoking is thought to decrease the enzymatic activity of histone deacetylases via oxidative stress.^154^

The link between microbiome and AMPA is furthermore intriguing, as secretory forms of ACPA, RF, and anti‐CarP are specifically present in RA and consist predominantly of the IgM isotype. Possibly, this observation suggests that these autoreactive IgM‐expressing B cells are continuously reactivated at mucosal surfaces, which may then contribute to the ongoing immune response.^155^ Future studies into the specific mechanisms linking microbiome with breach of tolerance toward posttranslationally modified proteins will further elucidate this intriguing topic.

### 9.6 Development of autoreactive B cells in RA

It is known that B cells, as opposed to T cells, are less rigorously controlled in terms of autoreactivity. The main checkpoints at which autoreactive B cells can be eliminated take place in the bone marrow during the early B cell development (central tolerance), followed by a checkpoint in the lymphoid tissue in the periphery (peripheral tolerance).^156^ At the central checkpoint, autoreactive B cells can undergo receptor editing, become anergic, or get eliminated (clonal deletion). Nevertheless, around 20% of the antibodies produced by mature naive B cells entering the periphery are still autoreactive^157^ and this number was shown to be even higher in RA patients.^158^ Already at the stage of new emigrant B cells and mature naive B cells, some clones from RA patients showed signs of reactivity toward citrullinated peptides and immunoglobulins, indicating impaired central tolerance checkpoints in these patients. This phenomenon has been linked to the genetic predisposing factor PTPN22, which possibly exerts its influence on the B cell activation threshold.^159^

At the periphery, recognition of an autoantigen generally leads to deletion of the B cell clone due to lack of T cell help. However, citrulline‐reactive B cells evidently receive T cell help and undergo subsequent maturation, as the vast majority of citrulline‐reactive B cells show signs of abundant somatic hypermutation (SHM)^49^ and are class‐switched toward IgG or IgA.^95^ In mice, the importance of T cell help for such autoreactive B cells is also highlighted by the following observation: When manipulated to acquire a certain specificity, murine IgM ACPA, but not anti‐influenza B cells became anergic.^160^ This implies that citrulline‐reactive B cells in the absence of T cell help do not undergo further differentiation, but get silenced by the immune system, at least in mice; the relevance of this observation for humans remains to be elucidated. The evident signs of T cell help in case of citrulline‐reactive B cells highlight the importance of investigating these presumably autoreactive T cells; this topic is discussed in more detail below.

Another important question considers the factors driving SHM in case of citrulline‐reactive B cells. During SHM, the BCR of a clone accumulates point mutations at a high rate. In the following selection process, BCRs in which point mutations resulted in higher affinity have a survival advantage. Surprisingly, ACPA do not seem to be of high affinity. Technically, ACPA affinity has not yet been conclusively measured due to technical challenges, but avidity maturation occurs only to a limited extent and only at around disease onset: Despite extensive SHM, ACPA remain of rather low avidity as compared to antibodies against foreign antigens.^161^, ^162^ Could it then be that the SHM in citrulline‐reactive B cells employs a more complex mechanism?

Recent studies have suggested that—in case of ACPA—clone selection during SHM may in fact be driven by the introduction of V‐domain *N‐*glycosylation sites.^49^, ^163^, ^164^ Intriguingly, these preliminary results indicate that V‐domain *N‐*glycosylation sites seem to be selectively introduced during SHM without unambiguously increasing affinity toward citrullinated antigens. Therefore, it appears that the *N*‐glycosylation sites may provide a different advantage. Various hypotheses have been put forward to explain the mechanism by which V‐domain glycans may influence B cell survival (Figure 2B). One of the proposed mechanisms suggests that V‐domain glycans impair self‐reactivity, thus providing the autoreactive B cells with the means to escape negative selection. Located at the antigen‐binding region of the BCR, a glycan could slightly decrease the affinity of the BCR for self‐proteins, which could result in salvaging of such “mildly” autoreactive clones. Such a mechanism was illustrated in mice where introduction of V‐domain glycosylation sites and subsequent decrease of affinity for a self‐protein seemed to give a selection advantage.^165^ Nevertheless, current results on the effect of ACPA V‐domain glycosylation on affinity suggest only minor changes,^46^, ^164^ which may not be enough for these autoreactive B cells to escape negative selection. Another proposed mechanism suggests that V‐domain glycans could influence the signaling threshold of BCRs. The balance between positive and negative signals ultimately determines B cell fate: for example, proliferation, differentiation, or apoptosis. Possibly, the V‐domain glycans would skew the balance toward positive, that is, proliferative, signals, thus explaining why B cells with V‐domain glycans would preferentially proliferate/survive. A similar mechanism is thought to play a role in follicular lymphoma B cells, which are also known to bear V‐domain glycans.^166^, ^167^ This could be explained by the interaction between sialylated V‐domain glycans and sialic acid binding lectins (Siglecs), expressed on the surface of B cells themselves or on other cells, including bacteria. Siglecs have been attributed various biologic functions including BCR signaling modulation.^168^, ^169^

Once developed, ACPA/AMPA B cells are thought to be continuously stimulated due to the ever‐present autoantigens. Evidence of continuous activation of the response can be derived from the fact that IgM ACPA are present in sera of patients with both early undifferentiated arthritis and established RA.^101^, ^102^ Moreover, ACPA IgM can (re‐)appear throughout the course of the disease.^101^ Together with the signs of abundant SHM in citrulline‐reactive B cells, these observations indicate that the activation of ACPA/AMPA B cells is an ongoing process.

Thus far, it has not been possible to discern a distinct pattern via which the AMPA response develops in the sense of antigen recognition: There does not appear to be a specific posttranslational modification that is recognized first and from which epitope spreading starts, although these investigations have yet to be performed for the most recently discovered AMPA targets (such as acetylated residues).^170^ The activation of certain AMPA B cells resulting in certain AMPA specificities may depend on the presence and abundance of modified proteins, certain modified sites, and the availability of T cells capable of providing help and inducing class‐switch recombination. Future studies may bring more clarity to the exact mechanisms involved in the breach of tolerance toward posttranslationally modified proteins and how this autoimmune response drives the development of RA.

### 9.7 Evolution toward RA: T cell involvement and the role of HLA

When discussing the involvement of T cells in RA development, it is important to review the current consensus regarding the stages of the disease evolution (Figure 1). It is unclear how tolerance to citrullinated residues is broken and which factors lead to the development of ACPA. As ACPA can be found years before the disease onset, the moment ACPA appear is often thought of as the “first hit” in the sense of the first detectable immunological event in the development of RA.

The presence of class‐switched ACPA (IgG, IgA) in predisease individuals indicates T cell involvement already at the stage of the “first hit.” However, little is known about the T cells that could be involved at this early stage. The fact that ACPA positivity in itself does not necessarily lead to RA development suggests that these first ACPA constitute a non‐autoimmune antibody response and that the T cells involved in providing help to ACPA‐producing B cells at this early stage would therefore be directed against non‐self‐antigens. Importantly, proper investigation of T cell involvement at the early pathophysiological stage represents a major challenge, as it requires identification of pre‐RA patients long before symptom manifestation.

More is known about the role T cells play at the later stages of RA development. The concept of the “first hit” presumes that the “second hit” or multiple subsequent “hits” would lead to disease onset. Among factors potentially involved in this “second hit,” there is substantial evidence pointing toward the importance of HLA. The maturation of the ACPA response is thought to be dependent on HLA‐SE‐associated T‐helper cells, which could potentially recognize the citrullinated antigen presented by the B cells. By providing help to citrulline‐reactive B cells, these SE‐associated (or even SE‐restricted) T cells would enable the predisease expansion and evolution of citrulline‐reactive B cells. However, the specificity of the T cells involved in this process and the exact mechanisms of their interaction with autoreactive B cells remain unknown. Overall, there is lack of consensus about involvement of T cells in RA pathogenesis, and two main scenarios have been suggested: Either the T cells are autoreactive and capable of inducing the response against self‐proteins, or they are in fact “healthy” and are specific against foreign, for example, bacterial antigens.^171^ In the latter scenario, cross‐reactivity of the pathogenic B cells recognizing both self and foreign antigens may be the means to receive the required T cell help. Considering the cross‐reactive nature of ACPA (discussed above in more detail) and the considerable challenge of identifying autoreactive T cells in RA patients, the second scenario seems more promising; however, future studies are required to elucidate the exact mechanism.

An interesting concept related to HLA involvement in RA pathogenesis is linked to the idea of protective HLA alleles. HLA‐DRB1*13 alleles were found to be protective in ACPA‐positive RA.^172^ This effect has been explained by specific properties of the DERAA sequence (located similarly to the “shared epitope”: positions 70‐74 of the beta chain), which has been proposed to function not as a presenting molecule, but as a self‐antigen. This “DERAA‐hypothesis” can best be formulated as follows: Peptides containing HLA‐derived DERAA can be presented by HLA‐DQ molecules. In the thymus of individuals with HLA‐DERAA alleles, this would lead to deletion of potentially DERAA‐reactive T cells.^173^ In individuals without DERAA‐containing HLA alleles, the DERAA‐reactive T cells would persist. These T cells could be stimulated by the DERAA sequence found in various bacteria. This can evoke an immune response that is cross‐reactive with vinculin, a human protein also carrying a DERAA sequence. Finally, vinculin is expressed in the synovium and can be detected in a citrullinated form in synovial fluid of RA patients. Since citrullinated vinculin can be recognized by T cells from HLA‐DERAA‐negative healthy individuals, this completes the “DERAA‐hypothesis”.^173^ The hypothesis suggests that T cells targeting microbial DERAA could provide help to B cells targeting citrullinated vinculin, thus resulting in activation of the ACPA response. Interesting insights regarding “DERAA‐hypothesis” have emerged from studies concerning microchimerism. Microchimerism (presence of > 1 genetically distinct cell populations in the same individual) occurs when an individual acquires allogeneic cells via various mechanisms; a typical example is bidirectional transfer of cells between mother and fetus during pregnancy. Intriguingly, opposite effects have been attributed to HLA‐DERAA‐positive cells, acquired via microchimerism, with some studies suggesting tolerizing properties, versus other studies proposing an immunizing or predisposing effect. On the tolerizing side is a report describing that children acquiring HLA‐DERAA‐positive cells from their mothers would be protected against RA, supposedly due to tolerization to the non‐inherited, but transplacentally acquired DERAA antigens, in the thymus during fetal development. On the other hand, a predisposing effect has been suggested to take place in mothers, as giving birth to HLA‐DERAA‐positive children may convey an increased risk of RA for DERAA‐negative mothers.^174^ In this setting, allogeneic fetal cells containing DERAA have been proposed to evoke an immune response that could be potentially cross‐reactive with DERAA‐containing vinculin. This was supported by the fact that DERAA‐microchimerism was found in more than 50% of mothers with RA and only in 6% of healthy mothers.^175^ Although further research is required to shed more light on the exact mechanisms involved in the effects of microchimerism, these findings are in line with the “DERAA‐hypothesis” described above.

Importantly, role of HLA may go beyond “shared epitope” and DERAA. Another recent study applied deep sequencing in order to identify novel genetic risks and identified two novel factors. One of them, that is, HLA‐DQα1:160 D, conferred strong susceptibility to ACPA‐positive RA and another one, HLA‐DRβ1:37N appeared to be protective.^176^ By using computational modeling, the authors suggested a possible role of these factors in antigen recognition by respective HLA‐typed T cells and their subsequent activation.

In conclusion, the data published so far suggest that in the evolution of RA, different T cells may be involved at different stages of the development of the ACPA response. During the predisease phase, T cells may be involved in the “first hit,” when they induce SHM and class‐switch recombination in citrulline‐reactive B cells resulting in production of ACPA IgG and IgA in the absence of symptoms. The specificity and properties of such T cells are at the moment subject of speculation. However, the evolution toward disease most likely depends on the “second hit,” during which HLA‐DRB1‐restricted T cells, and “shared epitope” and DERAA are likely to be involved in the B cell‐T cell interaction at this stage.

## 10 CONCLUSION

Extensive studies of RA pathophysiology and epidemiology have greatly expanded our knowledge of the disease development. Insights gained from the analysis of risk factors, such as smoking and the HLA “shared epitope” alleles, have fueled hypotheses about the development of autoimmunity. Findings regarding autoantibody responses to posttranslational modifications have instigated a new search for the possible inciting autoantigens and links with the microbiome. Despite these advances, some of the key questions regarding the breach of tolerance toward self‐antigens and the exact mechanisms involved in transition from predisease to disease onset remain to be elucidated. It appears that understanding the origins of the ACPA/AMPA response and the underlying causes of its unique properties, such as high V‐domain glycosylation, hold the promise of bringing our understanding of RA to the next level.

## CONFLICT OF INTEREST

We have no conflict of interest regarding this review.
